# Preparation and Evaluation of Transdermal Plasters Containing Norfloxacin: A Novel Treatment for Burn Wound Healing

**Published:** 2010-06-21

**Authors:** Kamal Dua, M. V. Ramana, U. V. S. Sara, D. K. Agrawal, Kavita Pabreja, Srikumar Chakravarthi

**Affiliations:** ^a^Department of Pharmaceutical Technology, School of Pharmacy and Allied Health Sciences, International Medical University, Bukit Jalil, 57000 KL, Malaysia; ^b^VIT University, Vellore 632014, India; ^c^DJ College of Pharmacy, Modinagar, Uttar Pradesh, India; ^d^Dr KN Modi Institute of Pharmaceutical Education and Research, Modinagar, Uttar Pradesh, India; ^e^Department of Life Sciences, School of Pharmacy and Allied Health Sciences, International Medical University, Bukit Jalil, 57000 KL, Malaysia; ^f^Department of Pathology, Faculty of Medicine, International Medical University, Bukit Jalil, 57000 KL, Malaysia

## Abstract

**Objective:** In an attempt for better treatment of bacterial infections and burn wounds, plaster formulations containing different concentrations of norfloxacin were prepared using polymers like polyvinylpyrrolidone and polyvinyl alcohol and evaluated for physicochemical parameters, in vitro drug release, antimicrobial activity, and burn wound healing properties. The prepared formulations were compared with silver sulfadiazine cream 1%, USP. **Methods:** Plaster formulations containing different concentrations of norfloxacin were prepared by solvent casting method using combination of polymers like polyvinylpyrrolidone and polyvinyl alcohol. These plasters were characterized for drug content, thickness, percentage elongation, tensile strength, in vitro drug release properties, and antimicrobial activity against various strains of aerobic and anaerobic microorganisms. The wound healing property was evaluated by histopathological examination and by measuring the wound contraction. **Results:** The in vitro release and in vitro skin permeation followed the first-order kinetics followed by diffusion as dominant release mechanism. In spite of the significant antimicrobial and wound healing effects produced by plasters, the observed values were less than the values obtained with silver sulfadiazine 1% cream (*P* < .05). Various histopathological changes observed during the study period (days 1, 4, 8, and 12) also supported the wound healing process. **Conclusion:** Based on the observed in vitro performances along with antimicrobial and wound healing effects, the 5% norfloxacin transdermal plasters could be employed as an alternative to commercial silver sulfadiazine 1% cream.

Ramakrishanan et al^1^ and Wang et al^2^ reported separately that anaerobic bacteria are the causative organisms for infection in around 15% of burn-infected patients. Huo^3^ reported that silver norfloxacin (NF) proved valuable in the treatment of burn wound infection caused by invading organisms, particularly by silver sulfadiazine-resistant strain of *Pseudomonas*.

Topical antibiotics can play an important role in prevention and treatment of many primary cutaneous bacterial infections commonly seen in dermatological practice like localized superficial infections due to surgery, injury, and abrasion.^4^ Topical antimicrobials help in preventing entry of microorganism into wound, which leads to fast healing of wounds. Quinolones belong to synthetic class of antimicrobial agents with potent antimicrobial activity which are effective orally and parentally for a wide variety of infectious diseases.^5^ Norfloxacin, a broad-spectrum fluoroquinolone antibacterial agent, is commonly employed in the treatment of urinary and genital tract infections.^6^^-^8 It is a hydrophilic fluoroquinolone with unique physiochemical properties such as low water solubility and partition coefficient.^9^^-^11

The objective of the present study was to prepare transdermal plasters containing different concentrations of NF and to evaluate burn wound-healing efficacy in comparison with marketed silver sulfadiazine 1% cream, USP.

## METHODS

Norfloxacin (Pfiscar India Ltd, Murthal, Haryana, India); polyvinylpyrrolidone (PVP), and polyvinyl alcohol (PVA) (SD Fine Chemicals, Mumbai, Maharashtra, India). All other chemicals used were of analytical grade, UV-spectrophotometer (Jasco V-530, Jasco Inc, 8649, Commerce Dr, Easton, MD 21601).

### Preparation of transdermal plasters

Transdermal plasters containing NF were prepared by casting method on mercury surface^12^ using PVP, PVA as polymers according to the composition given in Table 1. Polymer solution was prepared by heating a mixture of PVP and PVA (1:0.8) in water (about 40 mL) for 30 minutes, until a clear solution was obtained. The above ratio was selected on the basis of a pilot study. Weighed quantity of NF was dissolved in the above polymer solution and volume was made up to 100 mL with water. Four milliliters of the solution was poured onto mercury surface contained in a square glass mould of 4 cm × 4 cm (fabricated locally). The glass moulds were placed on a leveled surface and the solution was allowed to cool and solidify at ambient conditions for 24 hours. The moulds were kept in vacuum oven at 40°C, 400 mm of mercury for 48 hours.^13^ The dried plaster films were removed from the moulds and 0.5-cm edges were cut along all 4 sides. Five formulations containing varying concentrations of NF (NF-1 to NF-5) as given in Table 1 were prepared.

The plaster films NF-1 to NF-5 were subjected to in vitro release studies to select the plaster exhibiting the maximum release. In the selected formulation with maximum in vitro release, glycerin was incorporated (as plasticizer) in concentrations given in Table 1 and NF-6 to NF-8 were prepared in a similar way as described earlier. Based on the physical parameters such as flexibility, texture, and ease of fabrication, NF-6 with 0.1 g/100 mL of glycerin was considered as optimum concentration. In this optimized formulation, dimethyl sulphoxide (DMSO) was incorporated as penetration enhancer at 3 different concentrations (Table 1) to prepare NF-9 to NF-11. The effect of different concentrations of DMSO on the physical properties and release rate of the drug from the plasters was studied to select the best formulation. The final formulation NF-12 was prepared with the selected optimized concentration of the drug and the above ingredients.

### Characterization of transdermal plasters

#### Plasters were visually observed for color, clarity, texture, and flexibility

- Thickness^14^ of plaster films was measured using a digital vernier screw gauge (Ultra Science Aids, Bangalore, India) at different places of the plasters and average thickness was calculated.
- Weight^15^ of 6 individual plaster films of 3 cm × 3 cm was determined using an electronic balance with sensitivity of 0.1 mg (Ohause Corporation, Tokyo, Japan) and the average weight was calculated. The test was performed on films that were equilibrated under anhydrous calcium chloride in a desiccator for 24 hours.
- Folding endurance^15^ was determined by folding and opening the plaster film at the same place repeatedly for 250 times or until a break developed at the place of folding. The result was expressed as the number of times the plasters was folded to develop a break (if a break developed).

### Uniformity of weight

Six patches from different films (1 × 1 cm^2^) were weighed individually using digital balance (Ohause Corporation, Tokyo, Japan), and the average weight was calculated.

### Percentage elongation at brake and tensile strength^16^

Percentage elongation at brake was determined by using the tensile strength apparatus (fabricated locally). The plasters were cut into a size of 3 cm × 1-cm rectangular strips. One end of the strip along its length was clamped to the tensile strength testing apparatus and the other end was attached to a movable rod. The movable rod was attached to a pan with the help of a nonstretchable string through a pulley. Weights were carefully added onto the pan and increased gradually. The elongation of the plaster was determined by measuring the distance moved by the pointer on a graph paper after addition of the weight each time. The weights were added, until the plaster was broken. Percentage elongation was determined using the following formula:

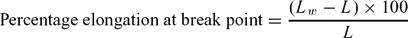



where *L* = initial length (in centimeter) of the plaster, and *L*_*w*_ = length (in centimeter) of the plaster when weight is added.

The tensile strength of the plaster was determined using the following formula:

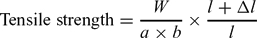



where *a* = width (cm), *b* = thickness (cm), *l* = length (cm) of the test plaster strip, Δ*l* = elongation (cm) at the break point, and *W* = weight (g) required to break the plaster.

### Percent moisture absorption

Weighed (*w*_1_) separately 6 plasters each of 1 cm^2^ and equilibrated over anhydrous calcium chloride for 1 week. Three plasters were exposed to ambient conditions of humidity (75%-85% RH, 28°C-32°C) and the other 3 to saturation humidity conditions (100% RH, 28°C-32°C) at room temperature for 2 days. Weight of each plaster (*w*_2_) was determined to calculate the weight gained by each plasters. Percent moisture absorption^17^ was calculated by using the following equation:

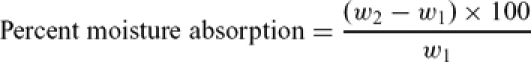



### Drug content and content uniformity

Transdermal plasters of each formulation were cut into pieces of 1 cm × 1 cm to determine the drug content and content uniformity.^18^ Six plasters, separately, were transferred into a glass mortar and triturated with 10 mL of 1% v/v acetic acid for 30 minutes. The contents were transferred into a 100-mL volumetric flask containing small amount of methanol and shaken continuously for 1 hour to extract the drug from polymer. The resulting solution was filtered through Whatman-1 filter paper and the drug content in the filtrate was determined by measuring the absorbance at 277.6 nm. Drug content per 1 cm^2^ of plaster was calculated by regression equation.

### Drug-polymer interaction studies

The drug-polymer interactions were studied by TLC (thin layer chromatography) analysis,^19^ Fourier transformed infrared (FT-IR) spectroscopy using Shimadzu FTIR-8400S Fourier transform infrared spectrophotometer,^12^ and differential scanning calorimetry (DSC) analysis using Shimadzu DSC-50 Thermal Analyzer.^20^

### In vitro diffusion studies

In vitro diffusion studies for all plaster formulations were carried out using Keshary-Chein type diffusion cell that comprised donor and receptor cells.^12^,^20^,^21^ Twenty percent v/v acetic acid was used as receptor media. The dialysis membrane (25 cm^2^) was soaked in water for a while and then for 2 hours in isotonic phosphate buffer solution, pH 7.4 (100 mL), prior to be mounted on the diffusion cell.

The membrane was tied securely to one end of the diffusion cell tube, the other end kept open to the ambient conditions. For this purpose, transdermal plaster of 1 cm^2^ was placed into the donor compartment of the diffusion cell that was immersed in the receptor compartment containing 20 mL of medium, which was continuously stirred using magnetic Teflon-coated bead. The entire system was maintained at 37 ± 1°C for 6 hours. An aliquot of 2 mL was withdrawn at 0, 1, 2, 3, 4, 5, 6, 7, 8, 12, 16, 20, and 24 hours. After each withdrawal, the diffusion medium was replaced with an equal volume of fresh diffusion medium maintained at 37 ± 1°C.

From the withdrawn aliquot of 2 mL, 1 mL was transferred in 100-mL volumetric flask, suitably diluted and the NF content was estimated spectrophotometrically at 277.6 nm. Average of 3 determinations was used to calculate the cumulative percent drug release at each time interval.

### In vitro skin permeability studies

This study was carried out for the best 3 formulations that exhibited the higher drug release through dialysis membrane. In vitro skin permeability studies were undertaken using Keshary-Chein diffusion cell^12^ in a similar way as described earlier but by using rat abdominal skin, instead of dialysis membrane. The rat skin was obtained from the abdominal portion of an albino rat after sacrificing the animal. The hair and fat were removed after treating the skin with 0.32 mol L^,1^ ammonia solution for 30 minutes.^18^ The skin was tied to the KC diffusion cell (donor cell) such that the stratum corneum side of the skin was kept in intimate contact with the release surface of the formulation in the donor cell. All experiments were carried out in triplicate.

### Kinetic analysis of release and skin permeability data

The release and skin permeability data from the best 3 transdermal plaster formulations were subjected to the kinetic analysis to establish the drug-release mechanism. The release and permeation data were fitted to zero-order,^22^ first-order,^23^ matrix (Higuchi matrix),^24^ and Hixson-Crowell equations.^25^

### Comparison of in vitro release and in vitro permeation profiles

The in vitro release and in vitro skin permeation profiles of the best among the 3 formulations in each case were compared for similarity. The in vitro release and in vitro permeation profiles of each of the 3 selected transdermal plaster formulations were compared for similarity with that of the commercial silver sulfadiazine 1% cream, USP. A model-independent approach was used employing a difference factor (*f*_1_) and a similarity factor (*f*_2_) as given in below equations, respectively^26^,^27^:

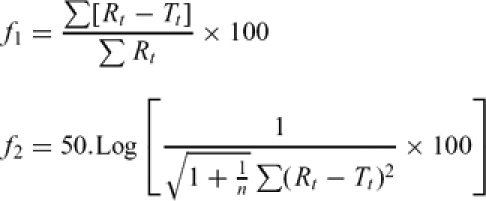



Where *R*_*t*_ and *T*_*t*_ are % dissolved for reference and test formulation at each time point and *n* is the number of time points dissolution profile. The time intervals used to study the *f*_1_ and *f*_2_ were up to 420 minutes.

The *f*_1_ value increases proportionally because of the dissimilarity between the 2 release profiles. If *f*_1_ value lies between 0 and 15 and *f*_2_ value of 2 drug release profiles is between 50 and 100, then these 2 drug profiles are considered similar. Value less than 50 indicates difference between the release profiles. The value of *f*_2_ = 50 reflects 10% difference; when value is >greater than 50, the difference between *R* and *T* is less than 10%.^28^

### Correlation of in vitro release and in vitro skin permeation data

The in vitro percent drug release from selected transdermal plaster formulations at different time intervals was plotted against the in vitro percent drug permeation at different time intervals. The *R*^2^ value was calculated after regression analysis. A linear relationship between the above 2 parameters would be indicated by the *R*^2^ values approaching to 1.^29^

### Stability studies

Stability studies on selected formulations were conducted according to International Conference on Harmonization guidelines. Drug-loaded transdermal plaster formulations were wrapped separately in aluminum foil and placed in polyethylene bag. The samples were stored at ambient conditions, 40 ± 2°C and 75 ± 5% RH for 6 months. Three samples of each formulation were withdrawn at 0, 30, 60, 90, and 180 days and analyzed visually for any changes in their physical characteristics and drug content as described earlier and the shelf life was determined.^30^

### Skin irritation test

Skin irritation was tested to find any allergic reactions caused by the application of topical/transdermal formulations. Six rabbits of either sex weighing 1.3 to 1.5 kg were used in this study. The abdominal hairs were removed by shaving. The shaved skin was cleaned with 70% alcohol and allowed to dry. Selected plaster formulation containing NF was applied on to the shaved skin of each rabbit and left in contact for 23 hours daily for 22 days. The skin was observed regularly for the study period for erythema (reddening of the skin), inflammation, contact dermatitis, or any change.^31^

### Microbiological studies

The antibacterial activity of various plaster formulations containing NF against various strains of aerobic and anaerobic microorganisms was evaluated by cup-plate method. *Bacillus subtilis*, *Staphylococcus aureus*, *Escherichia coli*, *Pseudomonas aeruginosa* (aerobic organisms), and *Bacteriodes fragilis* (anaerobic organism) were used for testing the antibacterial activity. Nutrient agar medium was used for aerobic bacterial cultures, and blood agar medium was used for *B fragilis*.

Plaster films containing NF were cut into circular pieces of 6-mm diameter and placed on the agar medium in plates. Silver sulphadiazine 1% cream (100 mg) was dispersed in 1 mL of water. Filter paper disks of 6-mm diameter were impregnated with the above dispersion and were placed on the agar medium plates. One sample was placed on each plate. The plates were prepared in triplicate. Plates with aerobic organisms were incubated at 37 ± 0.2°C for 24 hours under aerobic conditions, while *B fragilis* cultures were incubated under carbon dioxide atmosphere in an anaerobic jar at 37 ± 0.2°C for 48 hours.^14^ Inhibition zone diameters were measured with the help of zone reader (Digital Antibiotech Zone Reader, Effem Technologies New Delhi, India).^32^,^33^,^34^

### Burn wound healing studies

The experiments were carried out as per the guidelines of Animal Ethics Committee. Healthy Wistar albino rats weighing between 150 and 180 g were used for burn wound healing. The animals were divided into various groups, each group containing 6 animals. The untreated group was taken as control. The dorsum of each rat was shaved and the burn wounds were inflicted on overnight-starved animals under pentobarbitone sodium (6 mg/100 g, ip), anesthesia. A 2 cm × 2-cm metal cylinder was placed on the shaven back of the animals. Melted wax at 80°C was poured into the metal cylinder and the wax was allowed to solidify. Eight minutes after this or until the wax was completely solidified, the metal cylinder containing wax adhering to the skin was gently removed to inflict a distinctly demarked burn wound.^35^,^36^ In all cases, the burns were third degree, and the percentage was in 1 sample unit area according to the rule of nines, that is 9% of the body surface involved.

Selected NF transdermal plaster films and the marketed Silver sulfadiazine 1% cream USP (500 mg) were applied to the wound-inflicted areas of animals every day from the day 1 until day 12. The epithelialization period and wound contraction was observed in the study. The epithelialization period was monitored by recording the number of days required for eschar to fall off from the burn wound surface without leaving a raw wound behind. The animals were observed for wound healing by measuring the wound contraction (tracing the raw wound area on a transparent polythene paper that was retraced on graph paper (to assess the area) up to 12th day postwounding. The wound contraction was calculated as percentage of original wound size for each animal of a group.^37^

### Histopathological studies

The rats were anesthetized and the burned skin tissue samples were collected from rats (days 1, 4, 8, and 12) for histopathological examinations. The samples were fixed in 10% neutral buffered formalin and were cut into 5-µm sections and stained with hematoxylin and eosin (H&E) and examined by light microscopy for morphological changes. A histopathologist, blinded to the study, was assigned to analyze and grade the histological changes of each group.^37^,^38^

### Statistical analysis

Data pertaining to antimicrobial and burn wound healing activity were expressed as mean ± SD and the data were analyzed by one-way analysis of variance (ANOVA) with the Tukey test for multiple comparisons using Jandel Sigma Stat statistical software, version 2.0. In all the analysis *P* < .05 was considered as statistically significant.

## RESULTS

The average thickness of the plasters ranged between 143 and 160 µm. The tensile strength varied from 22 to 44 g/cm^2^. Among the various ingredients present in the formulations, PVP and glycerin were responsible for absorbing moisture. Drug loading varied from 91.1% to 97.6%.

### Drug-polymer interaction studies

A minute variations of *R*_*f*_ values of NF as pure drug (*R*_*f*_ = 0.27) as compared with that in transdermal plasters given in Table 2 indicated the compatibility of drug with PVP and PVA. This variation was expected to be of the normal experimental nature.

**Figure 1:**
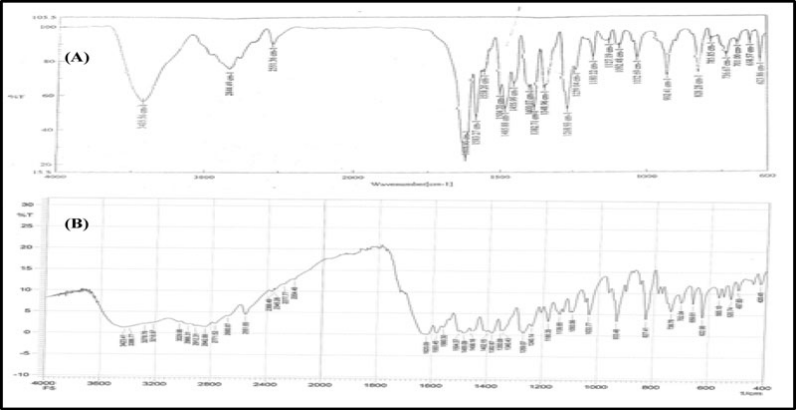
FTIR spectra of (A) Norfloxacin and (B) 6-month-old norfloxacin topical film (NF-11).

The drug was found identifiable by the presence of the characteristics bands. All the characteristic bands of NF were observed in the drug-polymer mixture. The findings are suggestive of the absence of any incompatibility between NF and polymers (Fig 1).

**Figure 2:**
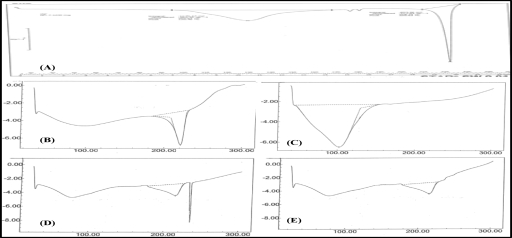
DSC Thermogram of (A) NF; (B) PVA; (C) PVP; (D) NF-PVP-PVA (PM); (E) 6-month-old NF topical film (NF-11). NF indicates norfloxacin; PM, physical mixture; PVA, polyvinyl alcohol; PVP, polyvinyl pyrrolidone.

The obtained DSC thermograms demonstrated a sharp melting endotherm at 227°C corresponding to the melting temperature of pure NF (Fig 2). It further indicated that the drug dispersed uniformly into the molten mass of polymers. The endotherm representing PVA was clearly distinguishable from the drug and PVP. The DSC further supported that NF was compatible with the polymers under study.

### In vitro release studies

**Figure 3:**
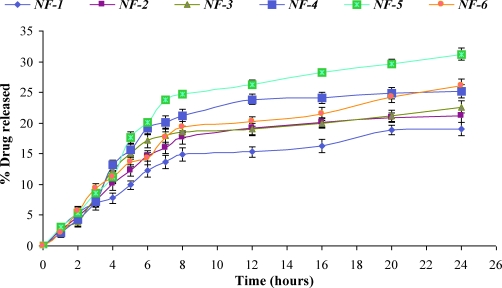
In vitro release profile of norfloxacin transdermal formulations (NF-1–NF-6).

**Figure 4:**
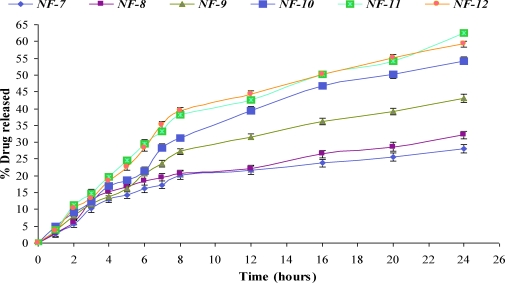
In vitro release profile of norfloxacin transdermal formulations (NF-7–NF-12).

The comparative release of NF from various formulations was shown in Figures 3 and 4.

### In vitro skin permeability

**Figure 5:**
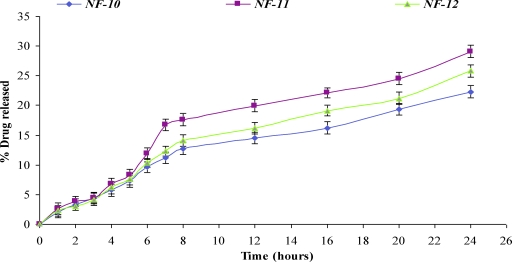
In vitro skin permeation profile of selected norfloxacin transdermal formulations (NF-10, NF-11, and NF-12).

The comparative release of NF from various selected formulations was shown in Figure 5.

### In vitro release and in vitro skin permeation—kinetics

The release of all plasters followed the first-order release kinetics, since the correlation coefficient (*R*^2^) for first order was higher in comparison to zero-order release. The results are in agreement with the previous investigations by Wahid et al (2008).^39^ When the values of the coefficients for “*t* (time)” were compared, the in vitro release of NF from NF-11 (*x* coefficient was 0.017) and in vitro skin permeation for NF-11 (*x* coefficient 0.0061) was found to be marginally higher in comparison to the other selected transdermal plasters.

### In vitro release–in vitro skin permeation correlation

As indicated by the *R*^2^ values higher than 0.97, a good correlation between the in vitro release and in vitro permeation was demonstrated for all 3 formulations. The lower values of coefficients of *x* coordinate further support a good correlation. However, the best correlation (*R*^2^ = 0.9844) was found for the NF-10.

### Comparison of in vitro release and in vitro permeation profiles

**Figure 6:**
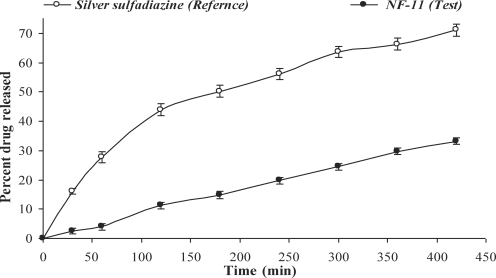
Analysis of in vitro release profile for *f*_1_ (difference factor) and *f*_2_ (similarity factor) of NF-11 with marketed Silver sulfadiazine 1% cream, USP.

**Figure 7:**
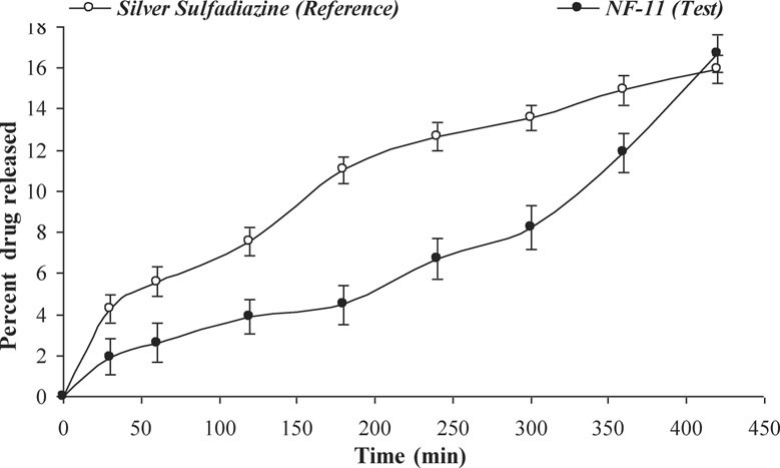
Analysis of in vitro skin permeation profile for *f*_1_ (difference factor) and *f*_2_ (similarity factor) of NF-11 with marketed Silver sulfadiazine 1% cream, USP.

The values of dissimilarity factor (*f*_1_) and similarity factor (*f*_2_) for the in vitro release and in vitro permeation are given in Table 3 and Figures 6 and 7.

### Stability studies

All plasters remained transparent and no visual differences in color or texture were observed. The degradation of NF in transdermal plasters followed first order, as the values of *R*^2^ for time versus log percent degradation graph were high. Degradation of all the three formulations followed almost to the same rate and extent. However, real-time studies for a period of 2 years are required to establish the stability of developed transdermal plasters.

### Skin irritation test

The skin irritation study indicated that neither the drug nor its components caused any edema or inflammation in or around the patch area during the period of study; however, reddening of the skin was observed after 7^*th*^ day, at the application site of transdermal plaster. However, the reddening of the skin disappeared after the discontinuation. Reddening of the skin may be due to the presence of DMSO in the formulation.

### Antimicrobial activity

**Figure 8:**
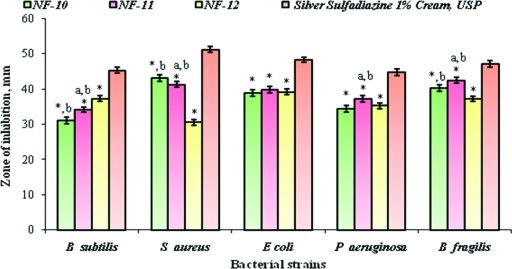
Comparative analysis of anti-bacterial activity of selected formulations of norfloxacin transdermal plasters with silver sulfadiazine 1% cream, USP. *P < .05 vs silver sulfadiazine 1% cream, USP. ^*a*^*P* < .05 vs NF-10; ^*b*^*P*< .05 vs NF-12.

As indicated by ANOVA statistics followed by the Tukey test, NF plaster (NF-11) had exhibited significant antibacterial activity against various strains of aerobic and anaerobic microorganisms that was in good agreement with silver sulfadiazine 1% cream, USP and the results obtained are shown in Table 4 and Figure 8.

### Burn wound healing activity

No mortality was observed in the animals during the study period. The mean period of epithelialization was found to decrease significantly in comparison to control (52.2 ± 1.2). However, no significant difference was observed in the mean values of NF-11 (27.0 ± 1.7) and silver sulfadiazine 1% cream, USP (25.9 ± 1.3).

**Figure 9:**
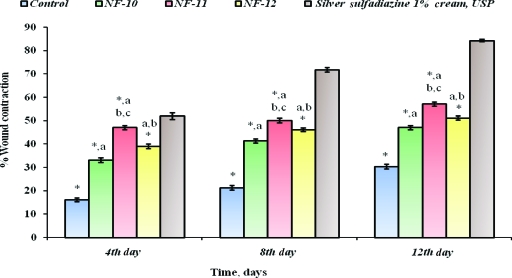
Comparative analysis of percent wound contraction of selected transdermal plasters of norfloxacin with silver sulfadiazine 1% cream, USP. *P < .05 vs Silver sulfadiazine 1% cream, USP. ^*a*^*P* < .05 vs control; ^*b*^*P* < .05 vs NF-10; ^*c*^*P* < .05 vs NF-12.

The difference between the mean percent burn wound contraction of the NF plaster treated animals as compared to control was found to be statistically significant. All the prepared formulations, including silver sulfadiazine 1% cream, USP, had shown wound healing activity (*P* < .05) in healthy male Wistar albino rats. Burn wound healing studies revealed a maximum percent wound healing of 57.15 ± 0.86% with NF-11 in comparison to silver sulfadiazine formulation available on the market, showing 84.34 ± 0.49% of wound healing within 12 days (Table 5 and Fig 9).

### Histopathological studies

**Figure 10:**
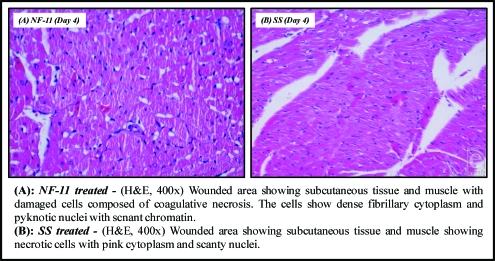
Microscopic appearance of burned skin on the 4th day.

**Figure 11:**
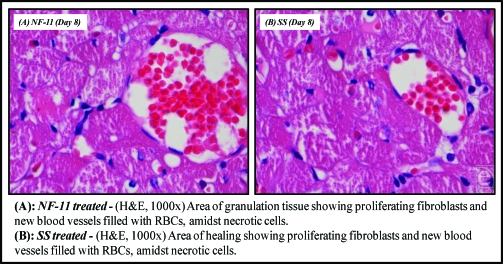
Microscopic appearance of burned skin on the 8th day.

**Figure 12:**
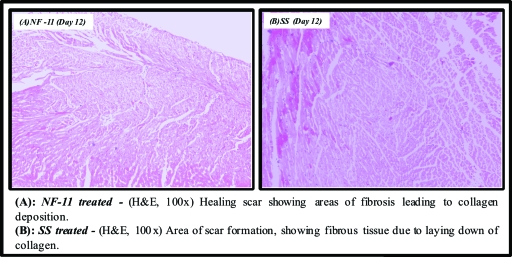
Microscopic appearance of burned skin on the 12th day.

The various histopathological changes observed during the study period (days 1, 4, 8, and 12) are tabulated in Table 6 and illustrated in Figures 10, 11, and 12.

## DISCUSSION

All the plasters were nonsticky. However, with increase in PVP, the characteristics of the plaster were found to change from nonsticky through semisticky to sticky. Based on this property, transdermal plaster containing 1.0 g/100 mL of PVP and 0.8 g/100 mL of PVA was finalized, which was observed to be nonsticky, tough, and easily moldable. Addition of glycerin in plasters NF-6 to NF-8 increased the thickness marginally, but the tackiness was comparatively enhanced. On the basis of these observations, 0.1 g of glycerin/100 mL was fixed as optimum (NF-6). The gradual increase in concentration of DMSO in the plasters NF-9 to NF-11 resulted in marginal increase in the thickness of the plasters. The weights were uniform on the basis of the considerations of dry weight of the ingredients.

On the basis of earlier studies, PVA:PVP in 1:0.8 was selected as a optimized ratio for the preparation of transdermal plasters. However, addition of glycerin and DMSO did not influence the tensile strength. Among the various ingredients present in the formulations, PVP and glycerin were responsible for absorbing moisture.

FTIR, DSC, and IR spectral studies performed on the films indicated no interaction between the drug and polymers. The drug was found identifiable by the presence of the characteristics bands. All the characteristic bands of NF were observed in the drug-polymer mixture.

Higher concentration of glycerin (above 0.1 g/100 mL) produced slight increase in release of NF but the films were found to be tacky and difficult to peel off from the surface. Glycerin was responsible for the increase in the flexibility of the film. As the concentration of DMSO was increased (NF-9 to NF-11), the release of NF increased from 43.12% to 62.66% at 24 hours (Fig. 4). The 62.66% release of drug from NF-11 was somewhat comparable with the 54.27% from NF-10, when DMSO concentration was increased from 2.0 g/100 mL to 3.0 g/100 mL. Although the maximum release was found with formulation NF-11 (62.66%), which contained 3% DMSO, but NF-10 (54.27% release) was regarded as the best formulation based on the physicochemical properties. The formulation NF-10 contained 2.0 g/{}100 mL of DMSO. PVP acts as nucleating agent that retards the crystallization of NF and hence enhances the release of the drug from matrix by sustaining it in an amorphous form.

With the increase in the concentration of DMSO, the in vitro skin permeability of NF was shown to be increased (Table 1). Maximum in vitro skin permeation of 29.12% of NF was observed from NF-11 (Fig 5), which contained a higher concentration of DMSO (3.0 g/100 mL). This higher in vitro skin permeability is reflective of DMSO as an effective permeability enhancer. The extent of in vitro release of drug through dialysis membrane was observed to be 20% to 30% higher when compared to in vitro skin permeability. Lower values of standard deviation are suggestive of the reliability and reproducibility of the experimental methods. The findings supported the skin penetration enhancement by DMSO.

The findings of this study are suggestive of the superiority of NF-10, NF-11, and NF-12 in terms of physical properties, in vitro release profile as well as skin permeation profiles. Further statistical analysis was done on the above 3 (NF-10, NF-11, and NF-12) formulations to determine the rate kinetics and mechanism of release. The release of all plasters followed the first-order release kinetics and the release mechanism, as indicated by the higher *R*^2^ values, was diffusion rate controlled following Higuchi model. As indicated by the coefficients of “square root *t* (time)” values the in vitro release of NF from NF-11 was higher (*x* coefficient was 13.75) whereas in vitro skin permeation was higher for NF-10 (4.857).

The value of *f*_1_ greater than 15 and *f*_2_ lower than 50 revealed that transdermal plaster (NF-11) was inequivalent to the commercial silver sulfadiazine 1% cream, USP. This implied that the polymer matrix retarded the drug release from the fabricated transdermal plaster. In terms of in vitro skin permeation, the *f*_1_ higher than 15 indicated dissimilarity of the permeation of drug from test transdermal plaster and reference formulation (marketed silver sulfadiazine 1% cream, USP) implying a corresponding slow permeation from test formulation. Although a similarity factor (*f*_2_) greater than 50 indicates the similarity of 2 formulations, yet the permeation profile (Fig. 7) along with higher *f*_1_ is sufficient to infer that the test formulation permeation is not similar.

## CONCLUSION

Norfloxacin plasters were easy to prepare and prevent microorganisms recess on the wound. The physical characteristics, such as thickness, average weight, consistency, elongation, tensile strength, moisture absorption, and drug content of NF plasters, were suitable. The polymer-drug interaction study did not show any incompatibility. The in vitro release of drug from the plaster followed the first-order kinetics followed by diffusion as a prominent release mechanism. The in vitro release was increased with increase in glycerin (flexibility enhancer) and dimethyl sulfoxide (penetration enhancer). In spite of the significant antimicrobial and wound healing effects produced by plasters, the observed values were less than the values obtained with silver sulfadiazine 1% cream (*P* < .05). The polymers in plaster retarded the release of the drug leading to a dissimilar release profile to that of the silver sulfadiazine 1% cream, USP.

Based on the observed in vitro performances along with antimicrobial and wound healing effects, the 5% NF transdermal plasters could be employed as an alternative to commercial silver sulfadiazine 1% cream. The therapeutic potential of these plasters may motivate researchers for its further exploitation for their commercial viability. This innovative mode of formulation can be employed for making burn wound healing process more effective.

## Acknowledgments

The authors are very thankful to M/s Pfiscar India Pvt Ltd, NH-1, 50th Km Stone to Delhi, Murthal, Haryana, India, and GCM Laboratories, Chandigarh, India for their generous gift sample of NF. The authors also place on record their thanks to U. P. Technical University, Lucknow, and VIT University, Vellore, India.

## Figures and Tables

**Table 1:** Composition of the TDDS formulations containing norfloxacin

Ingredients Formulation	NF (g)	PVP (g)	PVA (g)	Glycerin (g)	DMSO (g)	Water to (ml)
NF-1	1	2	1.6	…	…	100
NF-2	2	2	1.6	…	…	100
NF-3	3	2	1.6	…	…	100
NF-4	4	2	1.6	…	…	100
NF-5	5	2	1.6	…	…	100
NF-6	5	2	1.6	0.10	…	100
NF-7	5	2	1.6	0.20	…	100
NF-8	5	2	1.6	0.30	…	100
NF-9	5	2	1.6	0.10	1.0	100
NF-10	5	2	1.6	0.10	2.0	100
NF-11	5	2	1.6	0.10	3.0	100
NF-12	5	2	1.6	0.10	2.0	100

**Table 2:** TLC data of transdermal plasters containing norfloxacin

**Formulation**	**NF-1**	**NF-2**	**NF-3**	**NF-4**	**NF-5**	**NF-6**
*R*_f_*	0.26 (0.02)	0.27 (0.05)	0.25 (0.03)	0.27 (0.08)	0.26 (0.04)	0.27 (0.06)
**Formulation**	**NF-7**	**NF-8**	**NF-9**	**NF-10**	**NF-11**	**NF-12**
*R*_f_*	0.24 (0.02)	0.27 (0.04)	0.23 (0.05)	0.28 (0.06)	0.27 (0.02)	0.25 (0.04)

*Mean and standard deviation (in parenthesis) of 6 readings.

**Table 3:** Analysis of f_1_ (difference factor) and f_2_ (similarity factor) value of NF-11 with marketed silver sulfadiazine 1% cream, USP

	NF-11	
Formulation⇒	*f*_1_	*f*_2_
In vitro release	62.91	24.56
In vitro skin permeation	41.23	65.73

**Table 4:** Antimicrobial activity of transdermal plasters (n = 6)

	Inhibition Zone Diameter, mean (SD), mm				
Formulation	*B subtilis*	*S aureus*	*E coli*	*P aeruginosa*	*B fragilis*
NF-10	31.07*,† (0.97)	43.18*,† (0.93)	38.94* (0.92)	34.48* (0.93)	40.29*,† (0.88)
NF-11	34.13*,†,‡ (0.77)	41.29*,†,‡ (0.88)	38.80* (0.94)	37.15*,†,‡ (0.91)	42.51*,†,‡ (0.88)
NF-12	37.26* (0.86)	30.50* (0.72)	39.21* (0.78)	35.22* (0.79)	37.18* (0.75)
Silver sulfadiazine 1% cream USP	45.33 (0.78)	51.22 (0.86)	48.31 (0.72)	44.72 (0.98)	45.33 (0.78)

* *P* < .05 *Vs* silver sulfadiazine 1% cream, USP.

† *P* < .05 Vs NF-12.

‡ *P* < .05 *Vs* NF-10.

**Table 5:** Percent burn wound contraction of transdermal norfloxacin plaster*

	% Wound Contraction			
Formulations ↓ Days →	4^th^ day	8^th^ day	12^th^ day	Period of Epithelialization (days)
**Control**	16.13† (0.78)	21.21† (0.99)	30.42† (0.95)	52.2† (1.2)
**NF-10**	33.12†,‡ (1.06)	41.34†,‡ (0.96)	47.13†,‡ (0.89)	34.90†,‡ (1.5)
**NF-11**	47.10†,‡,§,∥ (0.93)	50.13†,‡, §,∥ (1.01)	57.15 †,‡,§,∥ (0.86)	27.00‡,§,∥ (1.7)
**NF-12**	39.12 †,‡,§ (0.99)	46.13†,‡,§ (0.75)	51.12†,‡,§ (0.90)	30.5†,‡,§ (1.6)
**Silver sulfadiazine 1% Cream, USP**	52.00 (1.45)	71.87 (0.98)	84.34 (0.49)	25.9 (1.3)

* Values in parenthesis indicate the standard deviation (*n* = 6).

†*P* < .05 vs Silver sulfadiazine 1% cream, USP.

‡ *P* < .05 vs control.

§*P* < .05 vs NF-10.

∥*P* < .05 vs NF-12.

**Table 6:** Comparative evaluation of histopathological observations of selected norfloxacin topical film with silver sulfadiazine 1% cream, USP

Day	NF-11	SS
**Day 1**	Ulceration and burns on the skin and subcutaneous tissue, coagulative necrosis	Presence of ulcer on surface due to injury, filled with slough and necrotic debris
**Day 4**	Extensive granulation tissue, reepithelialization, moderate scab formation, good proliferation, numerous inflammatory macrophages, and fibroblasts seen	Scab formed, beneath which a clot composed of fibrin, red blood cells, and platelets are formed. Inflammatory cells like macrophages and neutrophils migrate to the site
**Day 8**	Fibroblasts proliferation, collagen laying down, angiogenesis, neovascularization, wound contraction seen	Areas of fibroblast proliferation, new blood vessel formation, wound begin to contract
**Day 12**	Prominent wound contraction, collagen, and fibrosis are increased because of healing process resulting in prominent scar formation, cellularity reduced	Collagen is laid down significantly, inflammatory cells are reduced because of the healing process, and presence of fibrous scar is noticed
